# Leaping to Diagnosis: The Frog Sign as a Key Clue in AVNRT

**DOI:** 10.1002/ccr3.70248

**Published:** 2025-03-13

**Authors:** Andreas Mussigbrodt, Maria Herrera Bethencourt, Romain Vergier, Marinela Blidar, Alberto Alfie

**Affiliations:** ^1^ Department of Cardiology CHU Martinique (University Hospital of Martinique) Fort de France France; ^2^ Electrophysiology Section, Cardiology Division Hospital Nacional Posadas Moron Province of Buenos Aires Argentina

**Keywords:** ablation, AVNRT, cannon A waves, frog sign, slow pathway

## Abstract

The “frog sign,” characterized by prominent cannon A waves, is a critical diagnostic clue for AV‐nodal reentrant tachycardia (AVNRT). It was confirmed through electrophysiological study and was successfully treated with radiofrequency ablation.

## 1 Case Presentation

An elderly female presented with episodes of vertigo and palpitations. A “frog sign” was observed, characterized by prominent cannon A waves in the jugular and subclavian veins due to atrial contraction against a closed tricuspid valve (Video S1). Regularly beating cannon A waves are typically seen in AVNRT, less commonly in other supraventricular arrhythmias, tricuspid regurgitation, ventricular tachycardia, heart block, and pacemaker syndrome [1, 2]. The ECG revealed a regular, narrow‐complex tachycardia without visible P‐waves, raising suspicion for AVNRT. The diagnosis was confirmed by an electrophysiological study, and radiofrequency ablation was performed (Video S1). During follow‐up, no further symptoms were reported.

## Author Contributions

**Andreas Mussigbrodt:** conceptualization, data curation, formal analysis, investigation, methodology, project administration, writing – original draft, writing – review and editing. **Maria Herrera Bethencourt:** data curation, investigation, methodology, project administration, validation, visualization, writing – original draft, writing – review and editing. **Romain Vergier:** conceptualization, project administration, supervision, validation. **Marinela Blidar:** investigation, validation, visualization. **Alberto Alfie:** investigation, validation, writing – original draft.

## Consent

We confirm that written patient consent has been signed and collected.

## Conflicts of Interest

The authors declare no conflicts of interest.

## Supporting information


**Video S1.** Frog sign: Regular pulsations of jugular and subclavian veins, a typical sign of AVNRT. ECG shows regular narrow‐complex tachycardia consistent with AVNRT. EP study confirms the diagnosis. 3‐D mapping displays the right atrial anatomy with His bundle (yellow dots) and right and left inferior extensions of the slow pathway as ablation sites (red dots).

## Data Availability

The data that support the findings of this study are available on request from the corresponding author. The data are not publicly available due to privacy or ethical restrictions.
